# An overview of use, knowledge and perceptions of the Internet in Spain

**DOI:** 10.1016/j.dib.2018.06.015

**Published:** 2018-06-18

**Authors:** Daniel Aranda, Jordi Sánchez-Navarro, Leila Mohammadi

**Affiliations:** Faculty of Information and Communication Sciences, Universitat Oberta de Catalunya (UOC), Av. Tibidabo, 39-43, 08035 Barcelona, Spain

**Keywords:** Internet, Spain, Attitudes, Perception, Access, Consumption

## Abstract

The present data article offers a dataset about the use, knowledge, attitudes, and perceptions of the Internet in the entire of Spanish territory (16–65+). The data is achieved through biennial surveys in 2011 and 2013. This allows observing the evolution of assessments, the perception of use and penetration that different digital services have in the Spanish society. The paper includes some descriptive data that shows characteristic of internet access and Internet consumption among internet users and non-users over 16 years old.

**Specifications Table**

**Table:** 

Subject area	Social Communication
More specific subject area	Information and Communication Technology (ICT) Skills
Type of data	SPSS, Table, and Figures
How data was acquired	CATI
Data format	Raw and analysed data
Experimental factors	Data from 2011 are categorized into 10 main parts which are: Socioeconomic characteristics of the interviews; Internet use; Opinion on the use of the internet; Other activities; Internet use forms; Privacy on the internet; Social media; Online education; TV consumption and 15 M movement.
	Data in 2013 are categorized into 8 main parts which are: Access, use, and adoption; Regulation, control and policy; Impact of the Internet on the media; Internet and social connections; Security and privacy; Internet and education; Entertainment and digital leisure and Internet cultures: opinions, perceptions and attitudes
Experimental features	The data is achieved through biennial surveys in 201, May 31 - June 7, (*N* = 2100), and 2013, December 2–20, (*N* = 1600).
Data source location	Spain
Data accessibility	Data is within this article

**Value of the data**

• The present research offers an understanding about the use, knowledge, attitudes, and perceptions of the Internet in the entire of Spanish territory at the time when the impact of the use of different online services that shape the Net is becoming noticeable in all areas of the society.
• This dataset pursues to draw a map of access, consumption, and perception of the Internet from a biennial survey representative of the Spanish population (16–65+) from 2011 and 2013.
• This data allows observing the evolution of assessments and penetration that different digital services have in the Spanish society.
• The data allows for an initial comparison between Spain and other countries participating in the World Internet Project (WIP). Not all countries publish raw data of their inquiries, so an in-depth comparison is not guaranteed, but all countries participating in the project must publish reports on their main findings, so an initial comparison is always possible [5], [6].

## 1 Data

In this paper, we present two data sets. One collected in 2011 and the other in 2013 [1], [2]. Dataset collected in 2011: The main data file spreadsheet (data view) accompanying this article contains 2100 rows of data (representing one individual person per row) with the columns containing variables derived from responses to the survey of 2011. The survey includes 42 questions accompanying different possibilities for answer located in 195 columns in the data view sheet.

Dataset collected in 2013: The main data file spreadsheet contains 1600 rows of data (representing one individual per row) with the columns containing variables derived from responses to the survey 2013. The survey includes 35 questions which in the datasheet are categorized into 7 parts which are: videogames benefits and risks; ICT use; playing videogames; sociodemographic; data to calculate; data not to be calculated and original variables.

The questionnaire also accompanying this article as well as some graphs that shows characteristic of internet access in Spain.

## 2 Experimental design, materials and methods

The data shown and discussed in this paper refer to the Spanish territory although they are placed within the WIP, which is an international collaborative project that joins over two dozen nations in studies of the social, economic, and political implications of the Internet. So, this data can be compared with the other relevant and available data of other participant countries.

The questionnaire that has served as the basis of the field work for conducting the survey has been agreed with the rest of the WIP members [3], in order to gather a set of data comparable with the other countries. However, in this regard, the questionnaires can be considered in two different categories of questions: The WIP common questions that provide the comparable data and specific questions for Spain which don’t allow a comparative test.

Following countries are WIP partners who participated in the project in 2011 and 2013 and they have done the similar survey:

• 2011: New Zealand, Switzerland, Sweden, Mexico, Italy, Colombia, Canada, Australia, United Kingdom, Poland and United States (Organizer).
• 2013: China, United Kingdom, Poland, Switzerland, Sweden, Italy, New Zealand, Chile, Uruguay, Australia, Middle East (Qatar, Bahrain, Jordan, United Arab Emirates, Tunisia, Egypt, Saudi Arabia, Lebanon) and United States (Organizer).

For the collection of fieldwork data, a universe was considered, formed by the population over 16 years old, living in Spanish houses with fixed telephone lines. In 2011, the resulting sample was of 2100 interviews and, in 2013, of 1600 proportional by Autonomous Communities to the real distribution of the population.

The primary sampling units were the Municipalities, selected in a random proportional manner for each province. Secondary units were households, by random selection of telephone numbers. The last sampling units were the individuals, following a cross-stratification of sex, age and size of the municipality. The margin of error for the total sample is in 2011 of ± 2.13% and in 2013 ± 2.45%, for *P* = *Q* = 50% and under the assumption of maximum indeterminacy. The method of data collection was the computer-assisted telephone interview (CATI Bellview). The fieldwork was carried out between May 31 and June 7, 2011, and between December 2 and 20, 2013.

The questionnaire combines different models of questions, from closed dichotomies to the use of a five-level ordinal scale (in which 1 means: disagree/ not important and 5 means totally agree/ very important). Both questionnaires can be divided into two parts. The first part includes the general WIP questions (common questions). The second part includes specific questions for Spain. The following table shows the major thematic areas of the surveys.

Table 1 demonstrates the common WIP questions subjects which are comparable to other similar surveys, also it shows the Specific questions for Spain in which only, the last item of 2013 (Internet cultures: opinions, perceptions, and attitudes) allows a comparable exam with data of WIP Britain 2013 which has done by Oxford Internet Institute (OxIS).

**Table 1:** Major thematic areas of questionnaires; 2011 and 2013.

	**Common WIP questions**	**Specific questions for Spain**
**2011**	• Socioeconomic characteristics • of the interviews • Internet use • Opinion on the use of internet • Other activities • Internet use forms • Privacy on the internet	• Social media • Online education • TV consumption • 15 M movement.
**2013**	• Access, use and adoption • Regulation, control and policy • Impact of the Internet on the media	• Internet and social connections • Security and privacy • Internet and education • Entertainment and digital leisure • Internet cultures: opinions, perceptions and attitudes

**Fig. 1:**
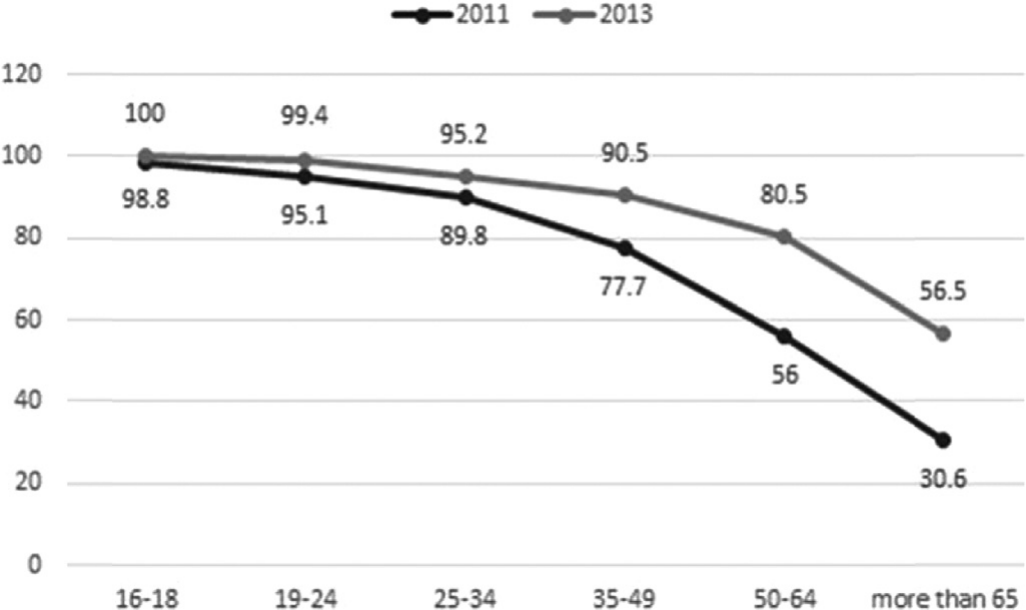
Internet users by age group (%). Source: Own elaboration, Base, 2011: 1455; Base, 2013: 1345.

**Fig. 2:**
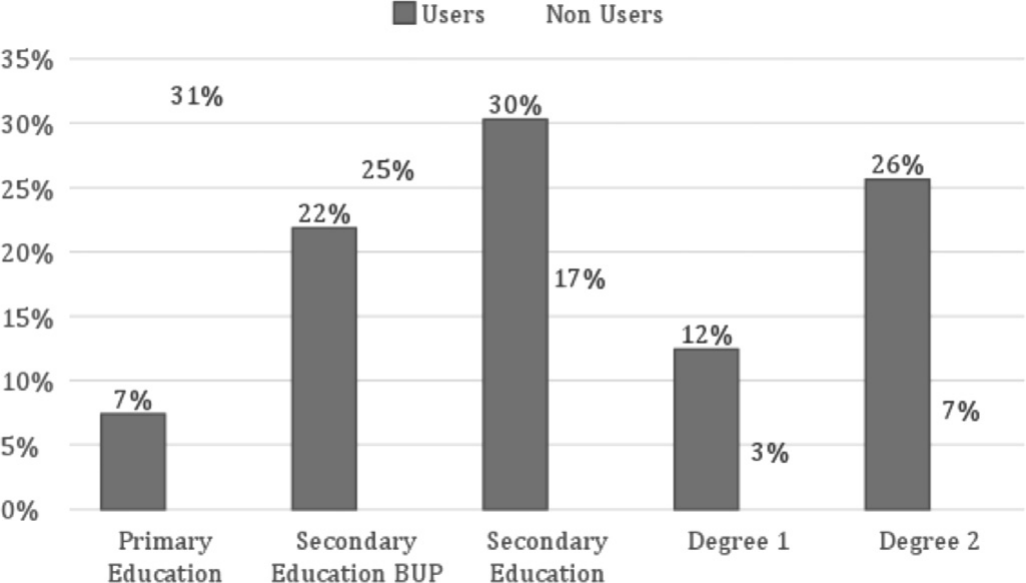
Education level of internet users and non-users in 2011. Source: Own elaboration, Base, 2011: 2100.

**Fig. 3:**
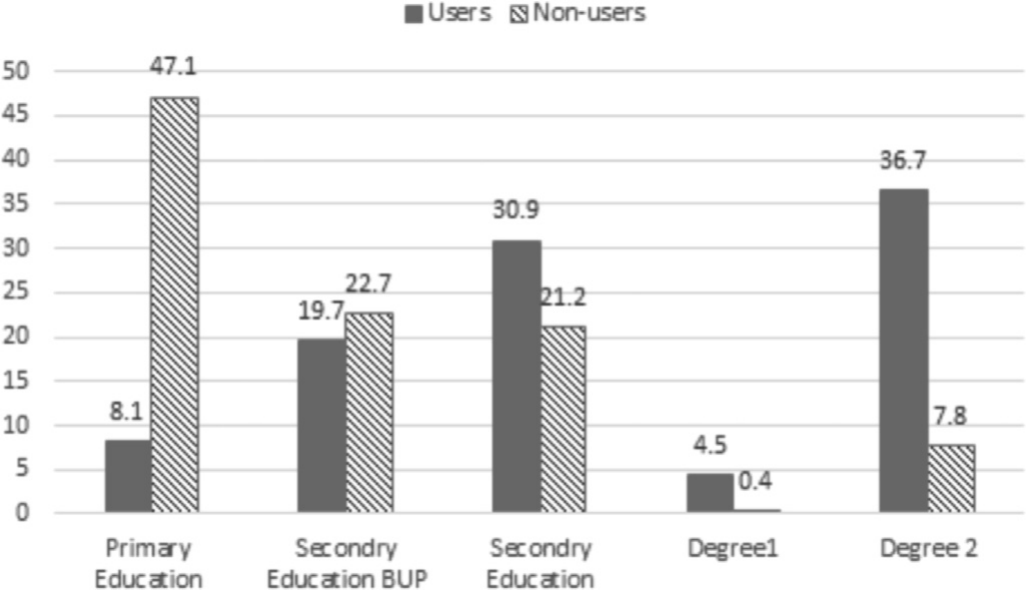
Education level of internet users and non-users in 2013 (%). Source: Own elaboration, Base, 2013: 1600 (user = 1345; non-user = 255).

**Fig. 4:**
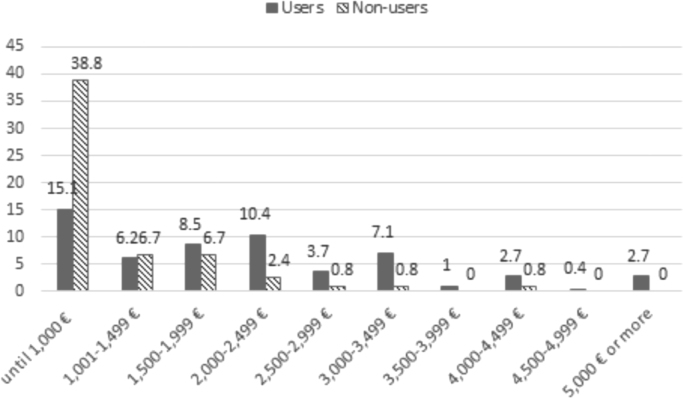
Household income level. Distribution according to monthly income (%). Source: Own elaboration, Base, 2011: 2100; Base, 2013: 1600.

Fig. 1, Fig. 2, Fig. 3, Fig. 4 show the characteristic of internet access in Spain:

## 3 Identifying cultures of the internet

To define and identify Internet cultures, the methodology applied in the study of WIP Britain 2013 and presented by Dutton and Blank from the Oxford Internet Institute (OxIS) [4] is transferred in WIP Spain 2013, and it is developed in different phases. First, a principal component analysis (PCA) is applied, using the same 14 variables that collected the British study and that reflect user attitudes towards the Internet collected through a Likert scale. After varimax rotation and Kaiser Normalization, four components showed eigenvalues above 1.0 that coincide with those registered in the study conducted by OxIS. These four components (cultural dimensions) are called: Enjoyable escape, Instrumental efficiency, Problem-generator, and Social-facilitator. This result is based on 1041 of Internet users, deleted missing values on the 14 Internet attitude variables.

These four cultural dimensions were further and previously analyzed in WIP Britain 2013. They identified five clusters or cultural groups among the Internet users using the following method.

The punctuation factors have been generated for each identified component and a hierarchical conglomerate analysis with Ward´s method and squared Euclidean distances has been applied based on the total of respondents [4]. We applied the same method in Spain for 1345 respondents. The missing values have been replaced by the means of the punctuation factors.

In order to characterize each group of Internet users (cultural groups), they have been positioned along the four cultural dimensions.

Each of these clusters allows identifying or recognizing a different type of culture that will be defined by the percentages of respondents that reach values in the above-average scoring factors, as shown in Table 2.

**Table 2:** Cultural groups. Cases whose punctuation factors are above the average per dimension. Source: Own elaboration, Base, 2013: 1345 users.

**Culture/ dimension**	**Cyber-moderates**	**e-mersive**	**Techno-consumersals**	**Adigital**	**Social-connected**
**Enjoyable escape**	45%	92%	21%	53%	17%
**Instrumental efficiency**	0%	59%	61%	70%	47%
**Problem-generator**	27%	69%	30%	14%	82%
**Social-facilitator**	12%	41%	2%	82%	68%
